# Supplementary material for: Corn Cob as a Green Support for Laccase Immobilization—Application on Decolorization of Remazol Brilliant Blue R
Source: Int J Mol Sci. 2022 Aug 19;23(16):9363. doi: 10.3390/ijms23169363 (PMC9409158; doi:10.3390/ijms23169363)
Supplement: Supplementary file 1 [file ijms-23-09363-s001.zip › ijms-1839573-supplementary.pdf]

International Journal of *Molecular Sciences*

MDPI

Article

Corn Cob as a Green Support for Laccase Immobilization and its application on Biodegradation of Remazol Brilliant Blue R

Supplementary Materials

Table S1. Effect on Immobilization Yeld and Recovered activity on immobilization of L_{Asp} on CC support.

pH	Immobilization Yield (%)	Recovered Activity (%)
**4**	18 ± 0.1	3.7 ± 0.4
**5**	29 ± 2.5	10.2 ± 1
**6**	53 ± 3.7	29.4 ± 5.8
**7**	75 ± 0.6	39.7 ± 1.3
**8**	41 ± 1.7	21.3 ± 2.8
**9**	14 ± 1.1	4.0 ± 1.6

Table S2. Effect of the time on Immobilization Yield and Recovered Activity in the immobilization of L_{Asp} on CC support.

Time (min)	Immobilization Yeld (%)	Recovered Activity (%)
**0**	0	0
**0.5**	11 ± 0.6	1.5 ± 1
**1**	26 ± 0.4	2.4 ± 0.5
**1.5**	48 ± 0.4	15 ± 0.2
**2**	74 ± 0.7	38 ± 0.2
**2.5**	73 ± 0.2	45 ± 1
**3**	73 ± 0.3	39 ± 0.1
**4**	74 ± 0. 1	40 ± 0.4
**6**	74 ± 0.6	39 ± 0.1
**8**	74 ± 1.5	39 ± 0.5
**12**	75 ± 0.5	40 ± 0.2
**24**	75 ± 0.3	39 ± 0.6

Table S3. Effect of ionic strength on Immobilization Yield and Recovery Activity at immobilization of L_{Asp} on CCsupport.

Ionic Strenght (mol . L^{-1})	Immobilization yield (%)	Recovered Activity (%)
0.01	83 ± 2.7	11.5 ± 1.6
0.025	83 ± 3	24 ± 3.3
0.05	76 ± 2	51 ± 0.4
0.1	75 ± 0.1	39 ± 0.6
0.2	66 ± 0.5	21 ± 4.9
0.25	62 ± 1.1	13 ± 1.2

Table S4. Recycling and determination of residual activity presented by immobilized L_{Asp} in the decolorization of the RBBR dye.

Number of cycles	Decolorization of RBBR (%)	Residual activity (%)
1	74 ± 1.6	99 ± 1
2	73.8 ± 3	97 ± 2.2
3	68.5 ± 1.5	92 ± 1
4	60 ± 2.1	87 ± 1
5	57.5 ± 1.6	70 ± 2.6
6	39 ± 2.1	55 ± 3
7	21 ± 1.2	31 ± 1
8	9.5 ± 2	16 ± 4.3
9	0.2 ± 0.6	6 ± 1.5

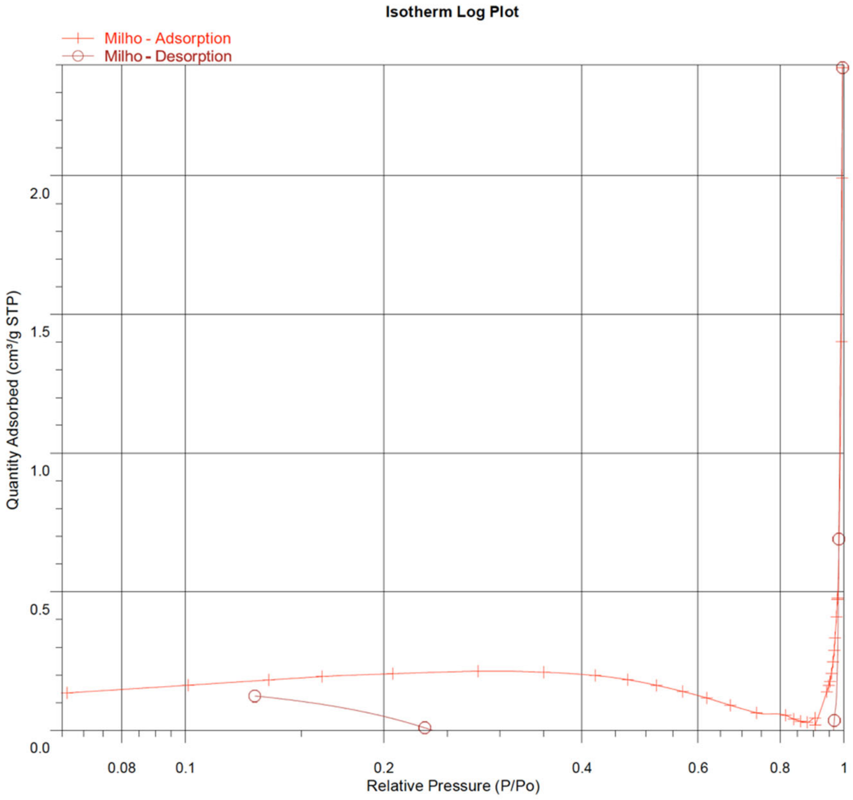

Figure S1. N_2 Adsorption and Desorption Isotherm of defatted corn cob in BET analysis (Log Plot).

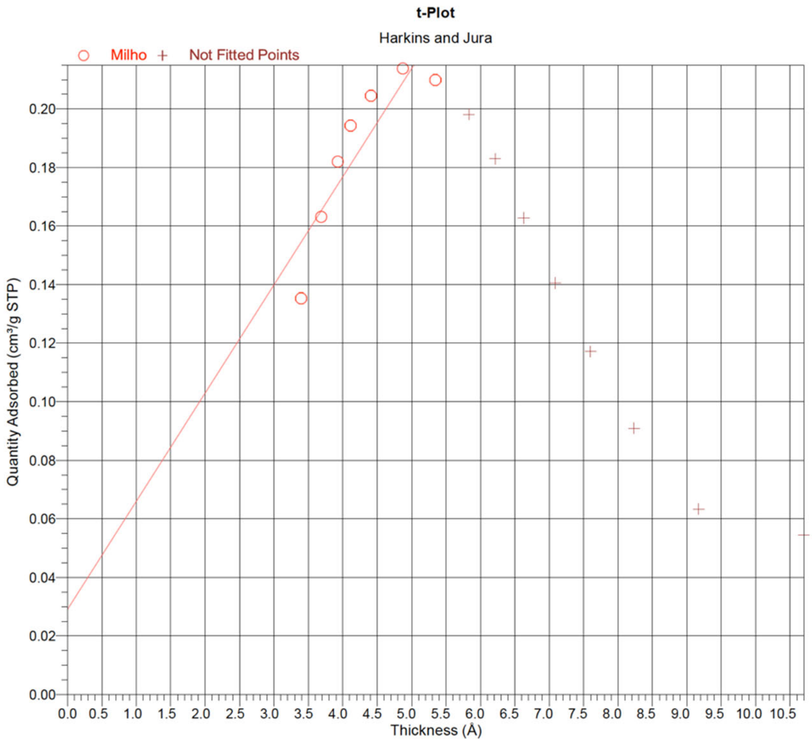

Figure S2. N_2 Adsorption and Desorption Isotherm of defatted corn cob in BET analysis (t-Plot).

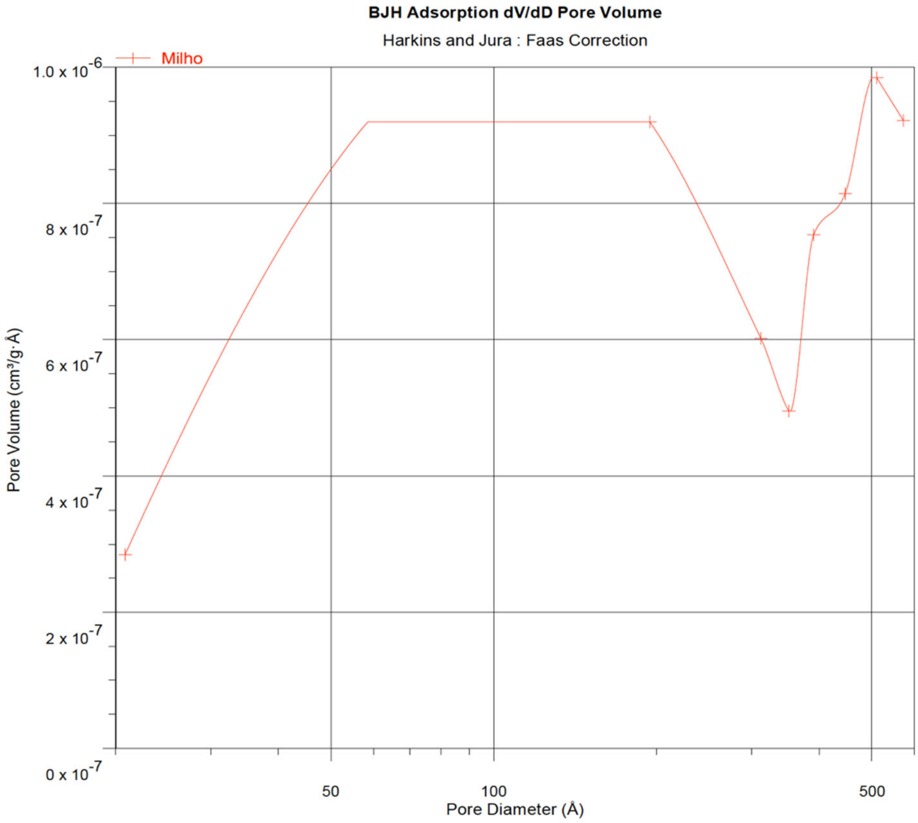

Figure S3. BJH Adsorption dV/dD Pore Volume of deffated corn cob.

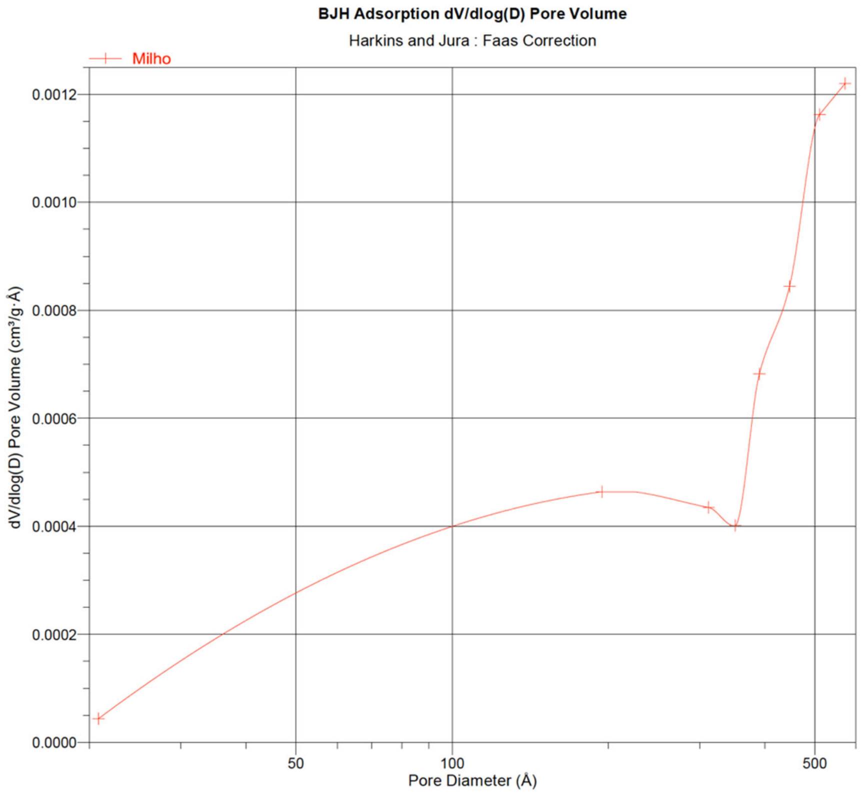

Figure S4. BJH Adsorption dV/dlog(D) Pore Volume.
